# Management and Treatment for Dysphagia in Neurodegenerative Disorders

**DOI:** 10.3390/jcm13010156

**Published:** 2023-12-27

**Authors:** Rumi Ueha, Carmel Cotaoco, Kenji Kondo, Tatsuya Yamasoba

**Affiliations:** 1 Swallowing Center, The University of Tokyo Hospital, Tokyo 113-8655, Japan; 2 Department of Otolaryngology and Head and Neck Surgery, Faculty of Medicine, The University of Tokyo, Tokyo 113-8655, Japan; mel_cotaoco@yahoo.com (C.C.); kondok-tky@umin.ac.jp (K.K.); tyamasoba-tky@umin.ac.jp (T.Y.); 3 Ear Nose Throat Head and Neck Surgery Institute, The Medical City, Metro Manila 1600, Philippines; 4 Tokyo Teishin Hospital, Tokyo 102-0071, Japan

**Keywords:** dysphagia, neurodegenerative disorders, Parkinson’s disease, multiple system atrophy, amyotrophic lateral sclerosis, Alzheimer’s disease, surgery

## Abstract

Patients with neurodegenerative disorders (NDDs) often experience functional dysphagia, which may involve dysfunction in a specific phase of swallowing or in the entire process. This review outlines the approach to dysphagia in the setting of NDDs. Distinguishing the etiology of dysphagia can be difficult, and it is important to always look out for signs pointing to NDD as the cause. Thorough diagnostic work-up is essential, and it includes a comprehensive history and physical examination, alongside swallowing function tests, such as fiberoptic endoscopic evaluation of swallowing, videofluoroscopic swallowing study, and high-resolution manometry. Management requires a multidisciplinary approach with a treatment plan tailored to each patient. This involves dietary guidance, swallowing rehabilitation, and surgery in cases in which improvement with rehabilitation is inadequate. Surgery may involve altering certain pharyngolaryngeal structures to facilitate swallowing and reduce the risk of aspiration (swallowing improvement surgery) or separating the airway and digestive tract while sacrificing laryngeal function, with the main goal of preventing aspiration (aspiration prevention surgery). Proper management stems from recognizing the impact of these disorders on swallowing and consistently finding ways to improve the quality of life of patients.

## 1. Introduction

Swallowing, also known as deglutition, is a sequence of processes that transports food from the mouth to the stomach. It includes the cognitive, preparatory, oral, pharyngeal, and esophageal phases [1,2]. Patients with neurodegenerative disorders (NDDs) may encounter disruptions in the seamless completion of swallowing movements, whether in specific phases or throughout the entire process [3,4]. In recent years, remarkable advancements in medical research have led to the elucidation of previously unknown pathologies and their causative genes. This progress has paved the way for the development of gene therapies and molecular-targeted treatments for NDDs [5,6]. However, without an accurate diagnosis of the underlying disease or pathology in patients, it becomes challenging to proceed with the evaluation of the condition and implementation of appropriate treatment.

Patients with a chief complaint of swallowing difficulties often consult general internists or otolaryngologists; however, physicians with limited expertise in managing swallowing disorders may face difficulties in discerning the etiology of dysphagia. There is a very small population of physicians who specialize in evaluating swallowing difficulties in patients with NDDs. Therefore, we have compiled information on swallowing disorders associated with major NDDs. Our aim is to enhance the understanding of dysphagia in NDDs among general physicians, otolaryngologists, as well as other health care professionals involved in dysphagia management. This review is based on our expertise in dysphagia management accumulated over 20 years and various previous publications. It outlines the approach to examining patients presenting with dysphagia as the primary complaint, including the diagnostic process and signs that may indicate the presence of NDDs. Additionally, this addresses the instructional aspects and surgical interventions pertinent to managing dysphagia associated with NDDs.

## 2. The Differentiation of Diseases Causing Dysphagia

Formulating differential diagnoses for swallowing disorders involves distinguishing among various conditions that can lead to impaired swallowing function. Dysphagia is categorized into organic swallowing disorders (static dysphagia) and functional swallowing disorders (dynamic dysphagia). Organic dysphagia results from structural abnormalities such as tumor lesions or inflammatory conditions extending from the oral cavity to the esophagus. It can also manifest as mechanical impediments to bolus passage due to irregularities in the passage or compression from the surrounding tissues. In contrast, functional dysphagia is characterized by normal anatomy but abnormal bolus transport function from the oral cavity to the esophagus. Other potential causes include eating disorders and psychiatric conditions [3,4]. Neurological disorders mainly present with functional dysphagia [7,8,9]. A comprehensive assessment of the patient’s symptoms and clinical findings is essential to accurately diagnose the underlying cause of dysphagia.

**Figure 1:**
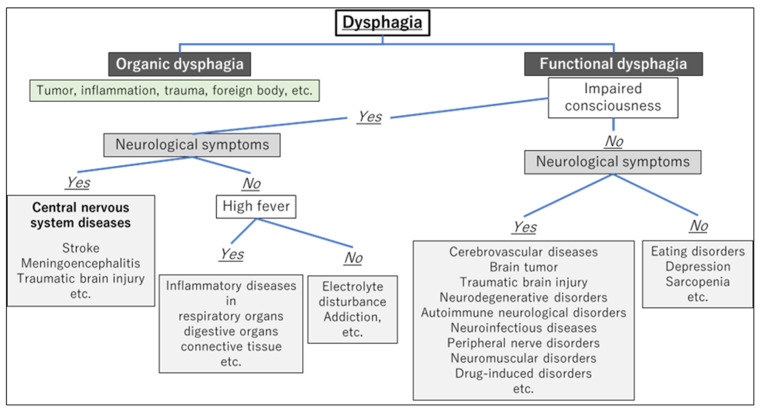
Flowchart for evaluating the underlying cause of dysphagia based on the presence of consciousness disorders.

**Figure 2:**
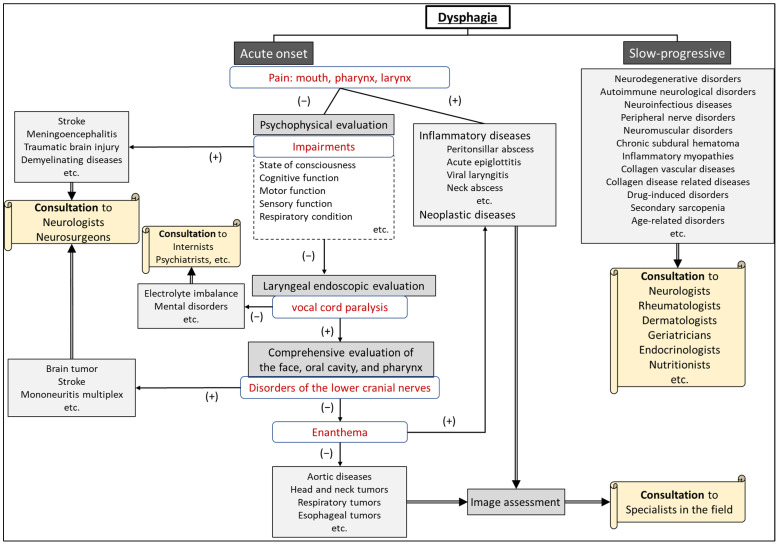
Flowchart for differentiating the etiology of dysphagia according to whether the onset is acute or slow-progressive.

After differentiating whether dysphagia is organic or functional, a flowchart for evaluating the underlying cause of dysphagia, based on the presence of consciousness disorders, is illustrated in Figure 1. A flowchart for differentiating the causative disease of dysphagia according to whether the course of dysphagia is acute in onset or slowly progressive is shown in Figure 2. In patient assessments, it is essential to prioritize a comprehensive interview, encompassing the current and past medical history, family background, medication usage, and other pertinent details, before proceeding to a meticulous evaluation of the oral and pharyngeal regions. Thorough psychosomatic assessments are highly important.

## 3. Neurodegenerative Disorders Causing Dysphagia

### 3.1. Representative Neurodegenerative Disorders Causing Dysphagia

Major neurodegenerative disorders (NDDs) that cause dysphagia include Parkinson’s disease (PD), progressive supranuclear palsy (PSP), spinocerebellar ataxia (SCA), multiple system atrophy (MSA), amyotrophic lateral sclerosis (ALS), spinal muscular atrophy (SMA), corticobasal degeneration (CBD), Alzheimer’s disease (AD), and Huntington’s disease, and so on.

This review describes dysphagia in patients with typical neurological disorders, namely, PD, MSA, ALS, and AD, along with their respective management approaches.

### 3.2. Parkinson’s Disease

PD is a neurodegenerative disorder characterized by symptoms such as resting tremor, muscle rigidity, and bradykinesia. More than 80% of patients with Parkinson’s disease develop dysphagia during the course of the disease; however, the degree of dysphagia does not necessarily correlate with the severity of PD [10]. In PD, all voluntary and involuntary motor processes in swallowing can be impaired: cognitive stage impairment due to depression and cognitive dysfunction [9], preparatory and oral stage impairment due to tremor and rigidity, pharyngeal stage impairment due to delayed swallowing reflex, decreased pharyngeal contractility, laryngeal elevation impairment [1,11], esophageal stage impairment due to upper esophageal sphincter (UES) dysfunction, and esophageal peristalsis impairment [12]. Furthermore, it should be noted that dysphagia can occur as a side effect of therapeutic medications in patients with PD [13]. Bilateral vocal fold movement disorders may also occur in patients with PD [14,15]. Tracheostomy, laser arytenoidectomy, or aspiration prevention surgery may be performed for progressive airway narrowing and severe dysphagia [14,15,16].

### 3.3. Multiple System Atrophy

MSA is a neurodegenerative disorder characterized by a combination of autonomic failure plus cerebellar syndrome and/or parkinsonism. MSA is categorized into two main subtypes: MSA-parkinsonian type (MSA-P), which is more like PD, and MSA-cerebellar type (MSA-C), which is associated with balance and coordination problems [17]. Dysphagia is a frequent and disabling symptom of MSA that occurs within five years [17,18]. Abnormalities in the oral and pharyngeal phases of swallowing, along with esophageal dysfunction and aspiration, occur in MSA and worsen as the disease progresses [17,19,20]. Bilateral vocal fold movement impairment (abduction disorder) occurs more frequently in MSA than in PD [21] and is characterized by an exacerbation of vocal cord dysfunction during sleep [22]. Depending on the progression of MSA, procedures such as tracheostomy or aspiration prevention surgery may be necessary [16,23].

### 3.4. Amyotrophic Lateral Sclerosis

ALS, also known as Lou Gehrig’s disease, is a progressive NDD. ALS affects the nerve cells in the brain and spinal cord. It specifically targets motor neurons, which are the nerve cells responsible for controlling muscle movements, including those used in talking, chewing, swallowing, and breathing [24]. Dysphagia is a significant and progressive symptom in ALS [25]. Dysphagia in ALS correlates significantly with bulbar onset and oral and pharyngeal swallowing impairment [25,26], while the esophageal phase is relatively preserved at the early stage of the disease [27]. Dysphagia contributes to malnutrition, dehydration, and aspiration pneumonia and accounts for a significant proportion of deaths in patients with ALS [25]. Early intervention and comprehensive management of dysphagia are crucial for addressing the risk of aspiration and its impact on the well-being of patients with ALS [28], and preventing aspiration is a critical aspect of managing ALS-related dysphagia [29]. Tracheostomy or aspiration prevention surgery may be performed because of respiratory deterioration or progression of dysphagia [16,29,30].

### 3.5. Alzheimer’s Disease

AD is a progressive brain disorder that affects memory, thinking, and behavior. As the disease advances, it can affect various aspects of a person’s health, including swallowing. In AD, the cognitive phase is most affected in feeding and swallowing behaviors. The prevalence of dysphagia is high in patients with AD, estimated at 84–93% [31,32]. Individuals often experience difficulties in coordinated eating movements, including challenges in understanding how to chew or swallow. Furthermore, muscle weakness and reduced sensation gradually impact the preparatory, oral, and pharyngeal phases of swallowing [33,34]. As swallowing rehabilitation and surgical treatments are rarely indicated for patients with AD, it is important to adjust their mealtime environment, positioning, and food and drink intake. Healthcare professionals should vigilantly observe individuals with AD for any indications of dysphagia and take necessary actions to ensure their safety and well-being while eating [35,36].

## 4. The Evaluation of Patients with Suspected Neurodegenerative Disorders

### 4.1. Patients with Neurodegenerative Disorders among New Outpatient Visits

In outpatient visits, some patients presenting with dysphagia or dysarthria as a chief complaint may have a background of NDDs. Patient data over approximately a decade (beginning in 2013) at the University of Tokyo Hospital showed that among 7910 patients attending the voice and swallowing clinic, 4973 (62.9%) had dysphagia, vocal fold movement disorder, or dysarthria. Of these, 1044 (13.2%) had already been diagnosed with NDDs (unpublished data). Among 3929 patients (49.7%) without a diagnosed underlying condition during the initial outpatient visits, 37 (1.0%) had suspected NDDs during otolaryngological examinations. Following referral to a neurologist, 19 patients (0.5%) were diagnosed with NDDs. This means that NDDs are hidden in the background of a small number of patients who present with dysphagia as their chief complaint.

### 4.2. Physical Signs and Oro-pharyngo-laryngeal Findings Suggesting Neurodegenerative Disorders

When the following findings are observed during medical interviews and physical assessments, it is recommended to proceed with the examination with consideration for NDDs:

- Weight loss;
- Gait disturbance/stumbling;
- Muscle weakness;
- Tremor;
- Reduced facial expression;
- Cognitive impairment;
- Respiratory dysfunction.

When the following findings are observed upon examination of the oral cavity, pharynx, and larynx, the possibility of neurodegenerative disorders should be considered in conjunction with other physical examination findings:

- Dysarthria;
- Speech impairment (hoarseness, low volume);
- Tongue atrophy and limited tongue movement;
- Velopharyngeal insufficiency (during articulation and/or swallowing);
- Vocal fold movement impairment;
- Reduced pharyngeal contraction;
- Decreased pharyngo-laryngeal sensation.

Dysarthria can be confirmed not only through pronunciation assessments, such as “/pa//ta//ka/,” “/ra//na/,” and “/ŋa//ŋa/,” but also through the repetition of tongue twisters when evaluating [37]. Tongue atrophy, tremors, and motor dysfunction are characteristic features observed in a variety of neuromuscular disorders [38]. Notably, tongue atrophy frequently manifests in conditions like ALS with bulbar involvement, muscular dystrophy, and severe myasthenia gravis. Tongue tremor is observed in conditions such as PD, essential tremor, and MSA-P [39,40]. When assessing tongue motor dysfunction, a finding of the mandible moving with the tongue is a compensatory maneuver for tongue motor impairment. Velopharyngeal insufficiency should be assessed during both swallowing and vocalization (/ka/, /ŋa/). Velopharyngeal insufficiency may be present during swallowing, even when open nasal speech is not present (velopharyngeal closure is possible during speech), and vice versa. ALS should be suspected when there is a discrepancy between velopharyngeal closure during swallowing and speech [41]. Velopharyngeal insufficiency can also occur in multiple sclerosis, Guillain-Barré syndrome, myasthenia gravis, muscular dystrophy, etc [42]. Vocal cord dysfunction (VCD) has been reported in various neurological disorders, including PD and MSA, and rarely in ALS and Guillain-Barre syndrome [43,44]. Reduced pharyngeal contraction and decreased pharyngolaryngeal sensation are commonly associated with a range of NDDs. In ALS, sensory disturbances are generally not evident until the disease has significantly advanced.

## 5. The Management of Dysphagia in Neurodegenerative Disorders

### 5.1. Meal-Time Management

Managing dysphagia in patients with NDDs involves providing support and guidance tailored to their current condition. Despite the frequent progressive nature of these disorders, there is the possibility of temporary improvement through pharmacological treatment, particularly in PD. Therefore, addressing swallowing difficulties in accordance with a patient’s specific conditions is advisable. Depending on the degree of the patient’s functional impairment, nutritional and swallowing guidance should include specific instructions regarding the following components: [45,46,47,48]

- Modifications of bolus texture (liquid thickness, swallowing diet)

For individuals with dysphagia, adjusting the physical properties of the diet according to swallowing function is important to lessen the risk of choking and subsequent aspiration pneumonia [49]. Increasing the bolus thickness has been identified as a means of lowering the risk of airway penetration. However, these adjustments in the physical characteristics of the bolus are associated with reduced palatability and an increase in pharyngeal residue, potentially amplifying the risk of post-swallow aspiration [50].

- Modifications of bolus size (the amount of food placed in the mouth at one time)

The modification of bolus size is a critical consideration in dysphagia management and should be tailored to the specific needs of the individual patient. Medical professionals may increase the bolus size to stimulate a swallow response or decrease it for patients who require multiple swallows per bolus. Larger bolus volumes have been associated with faster pharyngeal transit, while smaller volumes may be safer for swallowing in some populations [51,52].

- Feeding posture

Effectively addressing feeding posture in the context of dysphagia, especially in individuals with neurological impairments, such as those found in ALS, involves specific head and body positioning techniques to optimize the safety and efficiency of the swallowing process. These strategies aim to reduce the risk of aspiration and ensure a satisfactory nutritional intake. [28,53,54]

- Compensatory swallowing techniques, such as chin tuck, Supraglottic Swallow, and head rotation (see Section 5.2)

In addition, guidance should be provided based on the patient’s medical and physical condition, including adjustments to meal settings, thoughtful supervision, posture modifications, and the utilization of self-help devices such as spoons of different sizes and lengths.

### 5.2. Swallowing Rehabilitation

The establishment of short- and long-term goals for patients undergoing swallowing rehabilitation varies depending on whether the condition is a progressive disease with no established treatment or a disease with available therapeutic approaches. However, the key points in the management of all swallowing disorders are conservative treatment, pharmacotherapy, and rehabilitation (swallowing voice, and speech). Swallowing training includes indirect exercises performed without the use of food, such as ice massages to facilitate the swallowing reflex [55], head-lift exercises (Shaker exercise) to strengthen laryngeal elevation [56,57,58], respiratory muscle training [59,60], and neuromuscular electrical stimulation [50,61]. In contrast, swallowing training can also include direct exercises that involve the use of food, such as effortful swallows and multiple swallows. Certain compensatory techniques used during eating are also a very common component of swallowing training, such as the chin-tuck maneuver and head rotation. Optimal intervention entails a multidisciplinary approach, with collaboration among physicians, speech-language pathologists, nurses, dietitians, and other healthcare professionals [3,50,62].

▪ Indirect exercises
  - Facilitating the swallowing reflex: ice massage [55]

Ice massage is a technique designed to trigger the swallowing reflex by lightly rubbing and applying pressure to the posterior tongue, tongue base, velum, and posterior pharyngeal wall with an ice stick for 10 s.

  - Strengthening laryngeal elevation: resistance-based exercises and head-lift exercises (Shaker exercise) [56,57,58,63]

These exercises aim to strengthen the muscles involved in swallowing, particularly in patients with reduced superior and anterior movements of the hyolaryngeal complex. These include head-lift (Shaker) exercises, Mendelsohn Maneuver, effortful swallowing, and chin-tuck maneuver against resistance. Head-lift (Shaker) exercises involve lying flat on the back and lifting the head to look at the toes while keeping the shoulders down for 60 s and repeating this three times. The second part consists of a repetitive movement: lifting the head to look at the chin, lowering it to the bed, and repeating this 30 times for three sets.

  - Respiratory muscle training [59,60]

This exercise is used to strengthen the muscles of respiration and is weakened by various conditions including ALS, PD, and chronic obstructive pulmonary disease. This can also help improve speaking, swallowing, and coughing, as these functions use the related muscles.

  - Cough reflex exercise [63]

This exercise aims to strengthen the muscles involved in swallowing and enhance airway protection. This results in an improved closure of the larynx and enhanced coordination between swallowing and coughing to prevent aspiration.

  - Neuromuscular electrical stimulation [50,61]

Neuromuscular electrical stimulation is a noninvasive therapy that aims to improve the coordination, endurance, sensory feedback, and timing of the muscles involved in swallowing. This treatment is often used in combination with traditional treatments to improve swallowing.

▪ Direct exercises
  - Effortful swallow [64,65]

This technique aims to recruit more motor units during swallowing, increase muscle demand, and create a muscle-training/strengthening effect. It involves continuously exerting force on the neck while maintaining the larynx in the maximally elevated position during swallowing.

  - Multiple swallows [66]

Multiple swallows involves a series of repeated swallows to clear a single bolus from the oropharyngeal cavity. This aims to enhance the ability to modulate the timing, force, and coordination of the multiple muscles involved in swallowing.

  - Alternate swallows [63]

This technique involves repeated swallows that alternate between solid foods and liquids as the bolus. This process is repeated several times. The purpose of this approach is to facilitate the movement of food and liquid through the swallowing process, helping to ensure safe and efficient swallowing.

▪ Compensatory swallowing maneuvers [67,68]
  - Chin-tuck maneuver [69]

This maneuver helps redirect food and liquid from the airway, reducing the risk of aspiration. It entails the individual tucking their chin toward their chest before or during swallowing. This position reduces the space between the tongue base and the back of the throat and increases pharyngeal pressure to facilitate bolus movement.

  - Head rotation [70,71]

Head rotation during swallowing can be performed as a compensatory technique for individuals with dysphagia. Studies have shown that head rotation can improve swallowing in patients with unilateral oropharyngeal dysphagia. This maneuver helps redirect food and liquids away from the airway, reducing the risk of aspiration.

  - Supraglottic Swallow [72]

The Supraglottic Swallow is a technique to prevent aspiration during swallowing. It involves the voluntary closing of the vocal folds by holding one’s breath before and during swallowing, followed by an immediate cough after swallowing. The maneuver aims to safeguard against aspiration and clear any post-swallowing residue.

## 6. Surgical Intervention for Dysphagia

In some cases, despite the use of more conservative treatment strategies such as thickening liquids and altering food textures, patients still experience persistent aspiration. For these cases, surgical intervention becomes necessary to prevent potentially life-threatening complications [73,74,75]. Surgical procedures for patients with severe dysphagia can be categorized into the following three procedures, based on their purpose: (a) tracheostomy to create a route for suctioning aspirated material and secretions from the lower respiratory tract through the trachea; (b) surgeries aiming to improve pharyngeal swallowing while preserving speech function (swallowing improvement surgeries) [73,75]; and (c) surgeries dedicated to preventing aspiration despite the loss of speech function (aspiration prevention surgeries): [74]

- Tracheostomy

A surgical procedure to create an opening in the anterior trachea to facilitate respiration and to remove aspirated material and secretions.

- Swallowing improvement surgeries

Surgical procedures to modify pharyngo-laryngeal structures to facilitate swallowing and reduce aspiration, while preserving voice function.

- Aspiration prevention surgeries

Surgical procedures prevent aspiration of food and saliva into the airway while sacrificing vocal function.

Many medical facilities perform tracheotomies for the purpose of securing a respiratory and suctioning route, but not many perform “swallowing improvement surgery” or “aspiration prevention surgery” as surgical interventions for severe dysphagia. In particular, surgeries to improve swallowing function are performed only in a limited number of medical facilities, because they require thorough preoperative assessment and postoperative rehabilitation. The utilization of surgical therapies for dysphagia will likely differ depending on the healthcare policies and insurance systems in each country or region. 

### 6.1. Swallowing Improvement Surgeries

Swallowing improvement surgery is designed to restore the patient’s ability to swallow, while maintaining vocal and respiratory functions of the larynx. The surgery aims to create a morphology that facilitates easy swallowing and minimizes the risk of aspiration. It applies to patients for whom rehabilitation efforts have been undertaken, yet the improvement remains inadequate, and symptomatic aspiration is observed [74,75]. 

Generally, this surgery is conducted on patients, particularly those following a stroke, with the expectation of symptom stabilization in the future, while it is infrequently performed for progressive diseases. However, it may be considered for patients with progressive diseases in the following situations: (1) patients who strongly aspire for a temporary improvement in swallowing function, even while recognizing the progression of symptoms, and (2) if the advantages of surgical treatment surpass the potential risks [73,75,76,77]. This decision should be reached following thorough informed consent from both the patients and their families.

### 6.2. Surgical Procedure Selection in Swallowing Improvement Surgeries

Swallowing improvement surgery encompasses a variety of surgical procedures, and it has been observed that a single procedure often provides limited effectiveness. It is a common practice to combine multiple surgeries customized to the specific condition of the case. The critical factor in choosing surgical procedures is a thorough understanding of the state of swallowing dysfunction, and the selection of procedures is based on the specific type of impairment. This means that the decision on which surgical procedures to choose or combine is individualized for each case. In addition to extensive physical examination and evaluation of the airway, a comprehensive, site-specific assessment of swallowing status using swallowing function tests (such as fiberoptic endoscopic evaluation of swallowing, videofluoroscopic swallowing study, and high-resolution manometry) is essential for developing a treatment plan.

**Figure 3:**
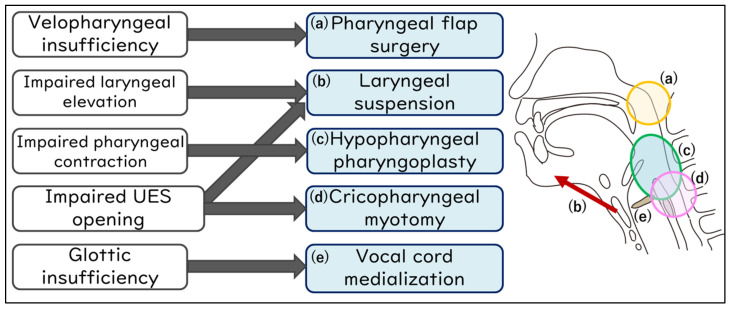
Swallowing improvement surgeries address specific sites in the swallowing mechanism.

Figure 3 details the association between the specific site of swallowing dysfunction and the corresponding surgical procedures. For velopharyngeal insufficiency, pharyngeal flap surgery is considered [78]; for impaired laryngeal elevation, laryngeal suspension (elevation) (with infrahyoid myotomy) [73,75,79]; for impaired pharyngeal contraction, hypopharyngeal pharyngoplasty [80]; for impaired UES opening, cricopharyngeal myotomy (with laryngeal suspension) [73,75,76,77]; and for glottic insufficiency, vocal cord medialization [81,82] is contemplated.

### 6.3. Aspiration Prevention Surgeries

Another surgical approach to severe dysphagia is aspiration prevention surgery, which involves separating the airway and digestive tract at the expense of sacrificing vocal function, primarily aiming to prevent aspiration [74]. While it does not guarantee postoperative oral intake, it is expected to enhance the quality of life of patients and caregivers by reducing suction frequency and preventing pneumonia associated with aspiration [74,83,84]. In essence, aspiration prevention surgery is not aimed at regaining swallowing ability at the expense of vocal function; rather, it is designed to prevent aspiration and improve quality of life. With the mention of aspiration prevention surgery in the medical guidelines for ALS and PD in Japan [29], there are increasing opportunities to perform this surgery for patients with PD, MSA, and ALS [23,29,85]. 

### 6.4. Surgical Procedure Selection in Aspiration Prevention Surgeries

**Figure 4:**
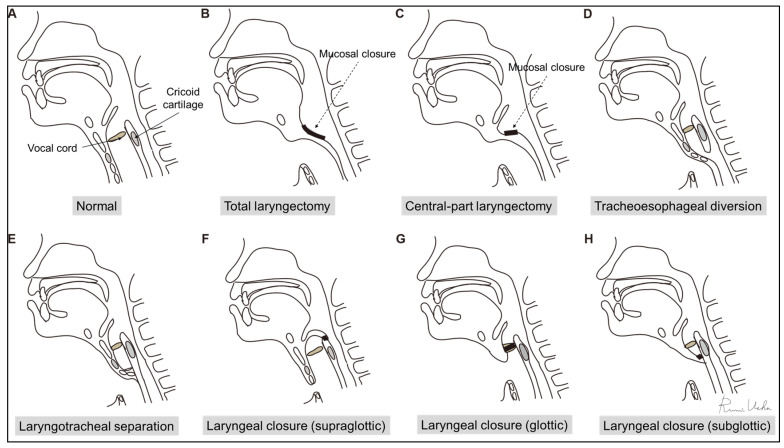
Aspiration prevention surgeries: (**A**) normal; (**B**) total laryngectomy; (**C**) central-part laryngectomy; (**D**) tracheoesophageal diversion; (**E**) laryngotracheal separation; (**F**) supraglottic laryngeal closure; (**G**) glottic laryngeal closure; and (**H**) subglottic laryngeal closure. (This figure has been modified from reference [50]).

Surgical techniques for preventing aspiration vary widely, encompassing procedures such as the removal of the entire larynx (total laryngectomy), partial removal of specific laryngeal regions (central-part laryngectomy), closure of the larynx (supraglottic closure, glottic closure, and subglottic closure), and separation of the airway at the tracheal level to preserve the larynx (laryngotracheal separation and tracheoesophageal diversion). These procedures are detailed in Table 1 and illustrated in Figure 4 [74]. While aspiration prevention surgeries can completely prevent aspiration, the postoperative feasibility of oral intake relies on the remaining swallowing function of patients. Even if oral intake is temporarily achieved after the surgeries, it will gradually become more challenging as the patient’s disease progresses. 

**Figure 5:**
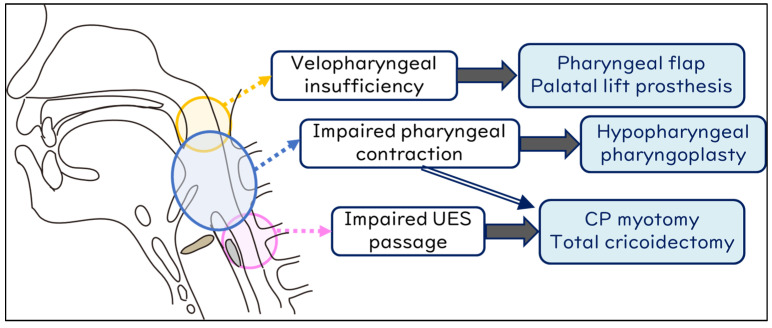
Procedures to facilitate oral intake after aspiration prevention surgery. UES, upper esophageal sphincter; CP, cricopharyngeal myotomy.

To assess surgical eligibility and select appropriate procedures, disease background, cognitive status, circulatory dynamics, respiratory condition, nutritional status, and cervicothoracic anatomy must be evaluated. All aspiration prevention surgeries can be performed under general anesthesia; however, for patients in which general anesthesia is not feasible, procedures that can be performed under local anesthesia should be selected. Furthermore, for patients desiring even slight oral intake post-aspiration prevention surgery, it may be advantageous to opt for a procedure that facilitates optimal dilation of the upper esophageal sphincter (UES), especially in cases where UES opening is compromised. In instances where there is difficulty in generating pharyngeal pressure, one may consider the simultaneous implementation of pharyngeal flap surgery or pharyngoplasty alongside aspiration prevention surgery (Figure 5). 

## 7. Discussion

Many physicians unfamiliar with NDDs will likely not suspect the possibility of NDD in patients who visit their clinic due to dysphagia, especially because the signs and symptoms can often be subtle. However, overlooking the possibility of this diagnosis can be detrimental to patients because they may not be referred to an appropriate specialist for further evaluation. Furthermore, it is impossible to provide appropriate advice and treatment for these patients without understanding the mechanism and progression of dysphagia caused by NDDs. Therefore, this review presents flowcharts (Figure 1 and Figure 2) for disease differentiation based on examination findings, which will help guide physicians in referring patients with dysphagia to an appropriate specialist.

Treatment strategies for patients with dysphagia due to NDDs include dietary guidance such as an adjustment of feeding methods and food properties, rehabilitation, and surgical treatment. While there is an abundance of literature available regarding dietary adjustment and rehabilitation, there are few articles discussing the surgical treatment of dysphagia outside Japan. Therefore, this review provides only a brief overview of the more conservative treatments for dysphagia and focuses more on the surgical management. For more than 20 years, many surgical procedures have been performed in Japan to improve swallowing function and prevent aspiration. These have been described and reported in many Japanese medical journals [74,84,86], but unfortunately, most have not been translated into English and are, therefore, inaccessible to the wider global medical community. In recent years, surgical treatments for dysphagia have become even more well-recognized in Japan, and guidelines for the treatment of NDDs now include surgical procedures as an option for patients with severe dysphagia [87]. Therefore, we have outlined the surgical treatments for dysphagia, including our past experiences, along with the illustrations above.

While it is inevitable that the approach to dysphagia management will differ between Japan and the rest of the world, it is our hope that this review article will promote the exchange of ideas and information between head and neck surgeons in Japan and other countries. In particular, our aim was to highlight surgical procedures as an option for treating dysphagia so that they will be more widely known and used worldwide. The details of the rehabilitation methods and surgical techniques are not included in this review article, and we refer the reader to more specialized books and articles for this information.

## 8. Conclusions

This review outlined the clinical examination approaches and management strategies [23] for dysphagia in patients with NDDs from the perspective of an otolaryngologist. In the management of swallowing disorders in patients with NDDs, essential points include not overlooking signs during routine clinical examinations, understanding the general course and physical manifestations of NDDs, recognizing their impact on swallowing, and consistently considering support for improving the quality of life of patients.

## Figures and Tables

**Table 1:** Aspiration prevention surgeries.

Aspiration Prevention Surgeries	
Surgeries to remove the larynx	Total laryngectomy
	Central-part laryngectomy
Surgeries to change the tracheal structure	Tracheoesophageal diversion
	Laryngotracheal Separation
	Tracheal flap method
Surgeries to close the larynx	Supraglottic laryngeal closure
	Epiglottic flap
	Vertical Laryngoplasty
	Transoral supraglottic closure
	Glottic laryngeal closure
	Subglottic laryngeal closure

## Data Availability

The datasets used and analyzed during the current study are available from the corresponding author upon reasonable request.
